# Supplementary material for: Piezomagnetism and magnetoelastic memory in uranium dioxide
Source: Nat Commun. 2017 Jul 24;8:99. doi: 10.1038/s41467-017-00096-4 (PMC5524652; doi:10.1038/s41467-017-00096-4)
Supplement: Supplementary file 1 — Supplementary Information [file 41467_2017_96_MOESM1_ESM.pdf]

File name: Supplementary Information
Description: Supplementary Figures, Supplementary Notes and Supplementary References

File name: Peer Review File
Description:

Supplementary Note 1: Piezomagnetism

The antiferromagnetic (AFM) transition at $T_N = 30.8$K in UO_2 is of the first order [1]. The axial thermal expansion measured along the body diagonal [111], $\varepsilon_a(T)$, is shown in Supplementary Fig. 1a along with the magnetic susceptibility M/H(T) between $T = 2$K and room temperature. The magnetization was measured in a magnetic field of 1kOe, in both field cooled (FC) and zero field cooled (ZFC) modes, with H applied along [111]. The AFM transition is clearly seen in both magnetic and lattice properties. The small upturn in the $M/H(T)$ curve (FC, blue line) below T_{N} is not expected for a simple AFM system. A somewhat similar feature was observed before in UO_2 under pressure, and attributed to

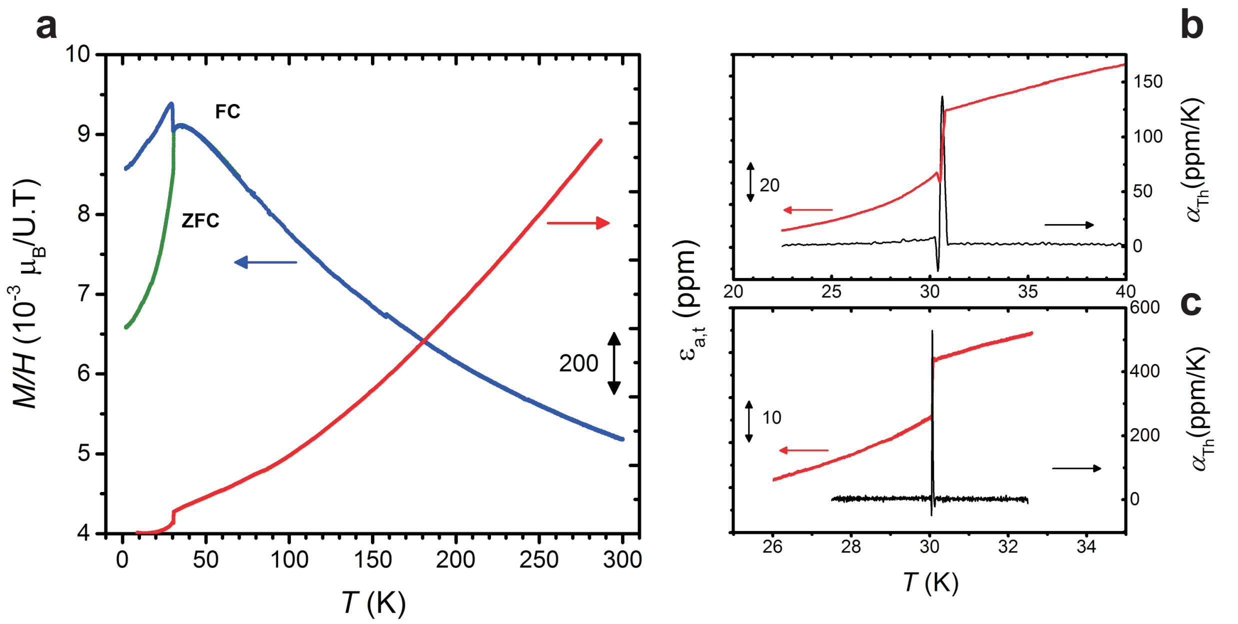

Supplementary Figure 1: **Thermal expansion and susceptibility versus temperature in** $\mathbf{UO_2}$.

(**a**) Zero field $\Delta L/L$ along [111] vs temperature (right y-axis) and magnetic susceptibility M/H in an external field of 1kOe in FC and ZFC modes (left y-axis), measured between 2K and room temperature. The antiferromagnetic transition is clearly visible. (**b**) and (**c**) Zero field $\Delta L/L$ of UO_2 vs temperature close to the AFM transition $T_{\mathrm{N}} \approx 30$K, measured along (**b**) and transverse (**c**) to the [111] direction, respectively. The small difference in the observed transition temperatures could be due to the poor thermal conductivity of optical fibers. The coefficients of thermal expansion α_{Th}(T) are ploted on the right hand side y-axes.

pressure-induced weak ferromagnetism [2]. Measurements in a field $H = 5$ kOe, on the other hand, reproduce ambient pressure results [2]. Supplementary Figures 1a and c show the axial and transverse magnetostriction $\varepsilon_{\mathrm{a}}(T)$, and $\varepsilon_{\mathrm{t}}(T)$, close to the AFM transition. The drop at T_{N} supports reduction in the volume of the unit cell in agreement with earlier conclusions [3, 4]. Also shown are the coefficients of thermal expansion $\alpha_{\mathrm{a}}^{T}(T)$, and $\alpha_{\mathrm{t}}^{T}(T)$ which are truly first-order like at T_{N}.

The observed linear magnetostriction (LMS) in the ordered state, precluded on the basis of time reversal symmetry considerations in most AFM materials, is strong in UO_2. The converse of the LMS phenomena is the piezomagnetic (PZM) effect. Borovik-Romanov [5] has considered the piezomagnetism from a phenomenological point of view, adding bilinear terms of magnetoelastic energy to the expansion of the thermodynamic potential per unit volume:

$$\Phi(T, \sigma, \mathbf{H}) = \Phi_0(T, \mathbf{H}) - \sum_{ijk} \Lambda_{ijk} H_i \sigma_{jk}, \tag{1}$$

where σ_{jk} are the components of the elastic strain tensor. If at least one term of this expansion remains invariant under the magnetic symmetry of the crystal, then the corresponding component axial Λ_{ijk} is not zero and hence the magnetization is given by

$$M_i = -\partial\Phi/\partial H_i = -\partial\Phi_0/\partial H_i + \sum_{jk} \Lambda_{ijk}\sigma_{jk}. \tag{2}$$

Thus, when a stress σ_{jk} is applied, a magnetic moment M_{i} linear in the stress is produced. It follows from Equation (1) that there exists also the LMS effect:

$$\epsilon_{jk} = -\partial\Phi/\partial\sigma_{jk} = \sum_{i} \Lambda_{ijk} H_i, \tag{3}$$

where ϵ_{jk} are components of the deformation tensor.

The possibility of existence of LMS/PZM in a system is thus related with a non zero tensor $\mathbf{\Lambda}$ invariant under the symmetry operations of the magnetic point group of the crystal. There are a total of 122 magnetic point groups which are obtained by including the time reversal symmetry operation R to the 32 point groups. It follows from Equation (3) that systems where the operation R appears in the magnetic point group as an independent operation does not present LMS/PZM. The reason is that since H_i changes sign against time reversal

symmetry and the components of the deformation tensor ϵ do not, then the third-rank axial tensor $\mathbf{\Lambda}$ must reverse sign upon a time inversion transformation R, which cannot therefore be a symmetry element of the magnetically ordered lattice. There are 32 such groups and 24 more where LMS/PZM cannot take place for similar reasons.

The subset of 66 magnetic point groups that can indeed display PZM/LMS were listed by Tavger [6] and Briss [7]; the space group $Pa\bar{3}$ $(m\bar{3})$, used to describe the AFM state in UO_2, is among them (see Supplementary Ref. [8]).

The magnetostriction tensor for the magnetic space group $\mathrm{Pa}\bar{3}$, point group $\mathrm{m}\bar{3}$ of UO_2 in Voigt's notation is:

$$\mathbf{\Lambda} = \begin{vmatrix} 0 & 0 & 0 & \Lambda_{14} & 0 & 0 \\ 0 & 0 & 0 & 0 & \Lambda_{14} & 0 \\ 0 & 0 & 0 & 0 & 0 & \Lambda_{14} \end{vmatrix} \tag{4}$$

The dependence of the strain tensor with the applied magnetic field in Supplementary Equation (3) can be explicitly written as:

$$\epsilon = \Lambda_{14} \begin{vmatrix} 0 & H_z & H_y \\ H_z & 0 & H_x \\ H_y & H_x & 0 \end{vmatrix} \tag{5}$$

For an applied field H along the [111] direction, the strain tensor can be diagonalized to give

$$\epsilon = \frac{H\Lambda_{14}}{\sqrt{3}} \begin{vmatrix} -1 & 0 & 0 \\ 0 & -1 & 0 \\ 0 & 0 & 2 \end{vmatrix} \tag{6}$$

Where the first two directions are perpendicular and the third parallel to the [111] direction. We thus obtain $\alpha_{\mathrm{t}} = -\Lambda_{14}/\sqrt{3}$ and $\alpha_a = 2\Lambda_{14}/\sqrt{3}$, hence it follows that $\alpha_{\mathrm{a}}/\alpha_{\mathrm{t}} = -2$.

Supplementary Note 2: The model Hamiltonian

We consider a classical Hamiltonian where the free degrees of freedom are the orientations of the magnetic moments of the four U atoms at the 4a positions in the $\mathrm{Pa}\bar{3}$ unit cell.

$$\hat{S}_i = S_0 \left[\sin\theta_i \cos\phi_i \; \hat{x} + \sin\theta_i \sin\phi_i \; \hat{y} + \cos\theta_i \; \hat{z}\right] \tag{7}$$

Interaction with the magnetic field

The Zeeman term gives the following contribution to the total energy:

$$\begin{aligned} H_{\rm Z} &= -g\mu_{\rm B}S_0\ \bar{H}.(\hat{S}_1+\hat{S}_2+\hat{S}_3+\hat{S}_4) \\ &= -g\mu_{\rm B}S_0\sum_{i=1}^{4}\ \sin\theta_i\cos\phi_i H_x+\sin\theta_i\sin\phi_i H_y+\cos\theta_i H_z \end{aligned} \tag{8}$$

If the magnetic field is applied along the (111) direction, one has:

$$H_x = H_y = H_z = \frac{H_0}{\sqrt{3}} \tag{9}$$

Magnetic anisotropy

The Jahn-Teller distortion stabilizes the 3-**k** magnetic order at $T < T_N$ producing a local anisotropy that can be written as:

$$H_A = -AS_0^2\ \sum_{i=1}^{4}\ (\hat{S}_i.\hat{v}_i)^2 = -A\frac{S_0^2}{3}\ \sum_{i=1}^{4}\ (\sin\theta_i\cos\phi_i\ u_{ix}+\sin\theta_i\sin\phi_i\ u_{iy}+\cos\theta_i\ u_{iz})^2 \tag{10}$$

here $u_{\rm i\alpha} = \pm 1$ (i $= 1,4$ and $\alpha = x,y,z$) are the sign of the projections along the Cartesian coordinates that specify the four directions of the magnetic moments in the 4a Wyckoff positions of the Pa$\bar{3}$ magnetic group.

This term is not enough to establish the relative orientation of the magnetic moments. A Heisenberg like interaction compatible with the symmetry operations of the magnetic group have to be included [10–12].

Heisenberg interaction

The Heisenberg contribution to the total energy, can be written as:

$$H_{SS} = -4JS_0^2\sum_{1\le i<j\le 4}\Big[S_{ix}(\hat{v}_i)S_{jx}(\hat{v}_j)+S_{iy}(\hat{v}_i)S_{jy}(\hat{v}_j)+S_{iz}(\hat{v}_i)S_{jz}(\hat{v}_j)\Big] \tag{11}$$

where the $S_{\rm i\alpha}(\hat{v}_{\rm i})$ are the three components of the magnetic moments with the z component along $\hat{v}_{\rm i}$.

Elastic energy

The elastic energy for a cubic crystal is:

$$H_{\text{el}} = \frac{a^3}{2}\Big[c_{11}(\epsilon_{xx}^2 + \epsilon_{yy}^2 + \epsilon_{zz}^2) + 2c_{12}(\epsilon_{xx}\epsilon_{yy} + \epsilon_{xx}\epsilon_{zz} + \epsilon_{yy}\epsilon_{zz}) + c_{44}(\epsilon_{xy}^2 + \epsilon_{xz}^2 + \epsilon_{yz}^2)\Big] \quad (12)$$

where $c_{11} = 389$ GPa, $c_{12} = 119$ GPa, $c_{44} = 60$ GPa, $B = 1/3\ (c_{11} + 2c_{12}) \simeq 207$ GPa, and $a = 5.47$Å. Only shear components of the strain can be kept in the expression above because they are the only components appearing in the magnetoelastic term (see below) :

$$H_{\text{el}} = \frac{c_{44}}{2}\ a^3\ (\epsilon_{xy}^2 + \epsilon_{xz}^2 + \epsilon_{yz}^2) \simeq 30.65\ \text{eV}\ (\epsilon_{xy}^2 + \epsilon_{xz}^2 + \epsilon_{yz}^2) \quad (13)$$

Magnetoelastic energy

To couple the elastic deformation to the magnetic order we include the magnetoelastic contribution (Supplementary eq. (1)) to the total energy :

$$H_{\text{me}} = -V \sum_{ijk} \Lambda_{ijk} H_i \sigma_{jk} \quad (14)$$

where $V = a^3$, $\mathbf{\Lambda}$ is given by equation (4), and the magnetoelastic contribution now reads :

$$H_{me} = -a^3\ \Lambda_{14}\ c_{44}\ \big[\epsilon_{yz} H_x + \epsilon_{xz} H_y + \epsilon_{xy} H_z\big] \quad (15)$$

where Λ_{14} is proportional to the staggered magnetization :

$$\Lambda_{14} = \frac{E}{c_{44}a^3} M_{\text{st}}, \quad (16)$$

$$M_{\text{st}} = \sum_{i=1}^{4}\ \hat{S}_i.\hat{v}_i \quad (17)$$

The minimization of the elastic and magnetoelastic contributions to the total energy gives :

$$\begin{aligned} \epsilon_{yz} &= \frac{E}{c_{44}a^3}\ M_{\text{st}}\ H_x \\ \epsilon_{xz} &= \frac{E}{c_{44}a^3}\ M_{\text{st}}\ H_y \\ \epsilon_{xy} &= \frac{E}{c_{44}a^3}\ M_{\text{st}}\ H_z \end{aligned} \quad (18)$$

The strain tensor for pure shear is

$$\epsilon = \begin{vmatrix} 0 & \epsilon_{xy}/2 & \epsilon_{xz}/2 \\ \epsilon_{xy}/2 & 0 & \epsilon_{yz}/2 \\ \epsilon_{xz}/2 & \epsilon_{xy}/2 & 0 \end{vmatrix} \tag{19}$$

In the case of a magnetic field along (111). $\epsilon_{xy} = \epsilon_{xz} = \epsilon_{yz}$, and the diagonalization of the strain tensor gives:

$$\epsilon_{111} = \epsilon_{xy}, \qquad \text{and} \qquad \epsilon_{100} = -\frac{\epsilon_{xy}}{2}. \tag{20}$$

Thus :

$$\epsilon_{111} = \frac{E}{\sqrt{3}c_{44}a^3}\ M_{\rm st}\ H \tag{21}$$

To have the experimental $\epsilon_{111} = \Delta L/L = 210\ 10^{-6}$ at 20 Tesla, $E \simeq 280\ 10^{-6}$ eV/Tesla.

Supplementary Note 3: Memory effect

On close inspection of Fig. 2a containing pulsed magnetic field data, and Fig. 2c (both in the main text of the paper) containing data taken in a slower superconducting magnet, we note a marked dependence of the coercive fields on field sweep rate. We further tested this behavior with measurements in two different pulsed magnets with sweep rates of 40 T/s and up to 10,000 T/s. The results are plotted in Supplementary Fig. 2, where we can see that the fastest sweep of 10000 T/s shown by curve 3 (red) results in a higher coercive field.

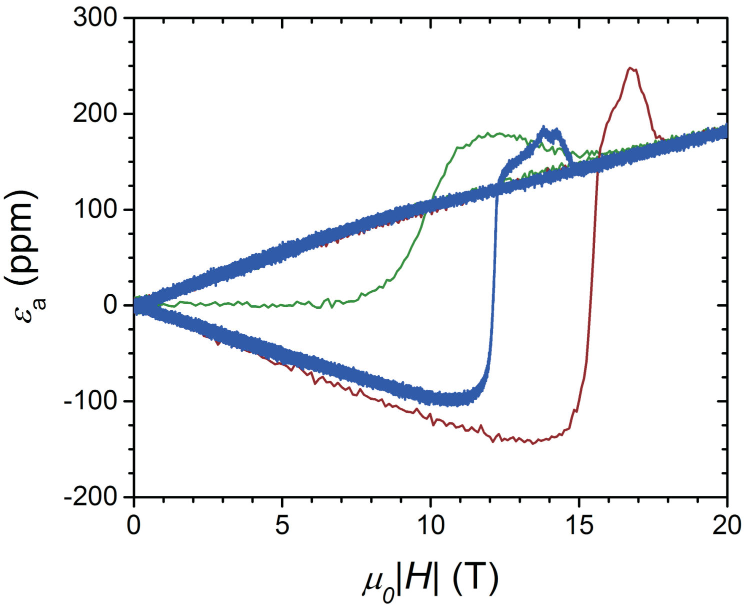

Supplementary Figure 2: **Memory effect in UO_2**.

$\varepsilon_a(H)$ for **H** along the [111] direction after zero field cooling the sample (green) compared with data obtained at an intermediate sweep rate of 40 T/s (blue) and at fast sweep rate of 10,000 T/s (red). This shows that the switching can be partial, allowing for tuning of $d\varepsilon/dH$. These characteristics make the gradual reorientation of magnetic moments a peculiar memory effect in UO_2.

SUPPLEMENTARY REFERENCES

[1] Jones, W.M., Gordon, J., & Long, E.A. The heat capacities of Uranium, Uranium trioxide, and Uranium dioxide from 15K to 300K. *J. Chem. Phys.* **20** 695 (1952).

[2] Sakai, H., Kato, H., Tokunaga, Y., Kambe S., Walstedt, R.E., Nakamura, A., Tateiwa, N., & Kobayashi, T.C., Pressure-induced weak ferromagnetism in uranium dioxide, UO2 *J.Phys.: Condens. Matter* **15** S2035 (2003).

[3] Brandt, O.G., & Walker C. T. Temperature depencence of elastic constants and thermal expansion for UO_2. *Phys. Rev. Lett.* **18**, 11 (1967).

[4] White, G. K., & Sheard, F. W. The thermal expansion at low temperatures of UO_2 and

UO_2/ThO_2. *J. Low Temp. Phys.* **14**, 445 (1974).

[5] Borovik-Romanov, A.S. Piezomagnetism, Linear Magnetostriction and Magnetooptic Effect *Ferroelectrics* **162**, 153 (1993).

[6] Tavger, B.A. Symmetry of Piezomagnetic Crystals *Sov. Phys. Cryst.* **3**, 341 (1958).

[7] Briss, R.R., & Anderson, J.C., Linear Magnetostriction in Antiferromagnetics *Proc. Phys. Soc.* **81**, 1139 (1963).

[8] Litvin, D. B., *Magnetic Group Tables* (International Union of Crystallography, 2013)

[9] Zvezdin A.K., *et al.*, Linear Magnetostriction and antiferromagnetic domain structure in dysprosium orthoferrite *Sov. Phys. JETP* **61**. 645 (1985).

[10] Santini, P., Carretta, S., Amoretti, G., Caciuffo, R., Magnani, N., and Lander, G. H., Multipolar interactions in f-electron systems: The paradigm of actinide dioxides, *Rev. Mod. Phys.* 81, 807-863 (2009).

[11] Carretta, S., Santini, P., Caciuffo, R., and Amoretti, G., Quadrupolar waves in uranium dioxide, *Phys. Rev. Lett.* 105, 167201 (2010).

[12] Caciuffo, R., Santini, P., Carretta, S., Amoretti, G., Hiess, A., Magnani, N., Regnault, L. P., and Lander, G. H., Multipolar, magnetic, and vibrational lattice dynamics in the low-temperature phase of uranium dioxide. *Phys. Rev.* B 84, 104409 (2011).
